# Evaluating industry attempts to influence public health: Applying an ethical framework in understanding commercial determinants of health

**DOI:** 10.3389/fpubh.2022.976898

**Published:** 2022-09-20

**Authors:** Paul Ndebele, Perrin Krisko, Imran Bari, Nino Paichadze, Adnan A. Hyder

**Affiliations:** Center on Commercial Determinants of Health and Department of Global Health, Milken Institute School of Public Health, George Washington University, Washington, DC, United States

**Keywords:** ethical framework, public health, commercial determinants of health, CDoH, industry practices

## Abstract

**Objectives:**

This paper explores industry influence on public health using a specific case study and applies an established ethical framework based on eleven principles to explore Commercial Determinants of Health (CDoH). It demonstrates an application of these principles to evaluate the ethical integrity of industry strategies and practices and their impacts on public health.

**Methods:**

Using eleven a priori, deductive, ethical principles as codes, this paper conducted an in-depth analysis of 19 e-mail chains and accompanying documents made publicly available through the Freedom of Information Act (FOIA) from U.S. Right to Know (USRTK) sent between Coca-Cola representatives, lobbyists, academics, and the International Life Sciences Institute (ILSI), founded by former Coca-Cola executives.

**Results:**

The three principles violated most frequently amongst the documents were consumer sovereignty (*n* = 22), evidence-informed actions (*n* = 21), and transparency (*n* = 20). Similarly, codes that featured most regularly across documents were transparency (13 out of 19 documents), consumer sovereignty (13 out of 19 documents), evidence-informed actions (9 out of 19 documents), and social justice and equity (9 out of 19 documents). All eleven principles were applied at least four times throughout the documents; however, responsiveness (*z* = 12), moral responsibility (*z* = 16), and holism (*z* = 30) were the least relevant to the data set.

**Conclusions:**

This case study of Coca-Cola demonstrates the usefulness of this ethics framework in reviewing actions of corporate actors in the promotion of products that are harmful to human health. It shows that the industry at times has low ethical integrity in their various strategies and practices to promote their products despite the negative impacts of these products on public health.

## Introduction

The core mission of public health is protection and promotion of the health of populations by identifying risk factors for disease, injury, disability, and death through the implementation of strategies aimed at reducing individual and group exposure to these risk factors (1). Public health policies are therefore mainly focused on prevention, accountability, and social justice (2). Corporate activities, including the promotion of hazardous products, such as sugary foods and beverages, have been criticized for contradicting the ideals of public health by promoting products and services that are harmful to health (3–6). Large amounts of salt, sugar, and fat in products made by food and beverage companies are major risk factors for diseases like obesity, type 2 diabetes, and other non-communicable diseases (NCDs) (7). Each year, diets high in sugar-sweetened beverages cause 242,219 premature deaths worldwide; nearly 40% of these deaths occur in low-middle income countries (LMICs) (8).

Commercial Determinants of Health (CDoH), which are factors that stem from profit maximization and influence public health, have contributed significantly to NCDs or “lifestyle” health outcomes (9). As participants of society, corporate entities serve a dual role, creating a conflict of interest between their objective to maximize profit and their duty to consumers. On one hand, corporations are expected to fulfill their responsibility to the public by negating harm onto others, opposing false information, and promoting public goals (10). On the other hand, they must fulfill their private interests as well by pursuing business interests, maximizing profits, market sharing, and obtaining returns on investments (9). These imperatives are reinforced by law, tradition, and ideology (10). When the corporation must decide between pursuing its public service vs. private profit motives, a conflict of interest may arise.

The ways in which corporations work often leads to conflicts between profit-generation and public health, hence the emergent field of CDoH (10). CDoH include strategies and approaches exploited by the private sector to promote products and choices that are detrimental to health (11). In the face of these powerful corporate actors, consumers, policymakers, and decision makers often face constrained choices. Therefore, it is crucial to utilize an ethical framework to evaluate the actions and strategies of corporate actors regarding products harmful to health. In 2021, the top five highest earning beverage companies in the world included Coca-Cola Company (Coca-Cola) (12, 13). Coca-Cola launched its sales on an international scale in the 1920s and has experienced widespread commercial success since then (14).

This paper applies an ethical framework for CDoH based on eleven principles to a case study implicating Coca-Cola (9). Through a rigorous literature search and consultations with experts in the field of CDoH, Paul et al. identified these ethical principles (9). The eleven ethical principles for CDoH include: moral responsibility, non-maleficence, social justice and equity, consumer sovereignty, evidence-informed actions, responsiveness, accountability, appropriateness, transparency, beneficence, and holism (9). This paper demonstrates ways in which these principles may be used to evaluate the ethical integrity of industry strategies and practices and their impacts on public health.

## Methods

The paper tests the usefulness and applicability of this ethics framework by evaluating Coca-Cola's attempts to influence public health. A series of e-mails exchanged between Coca-Cola staff and affiliates from 2014 to 2015 were requested and obtained, covering a range of topics from the state level to international including discussing strategies to coerce World Health Organization representatives to oppose sugar taxes, and arranging private meetings with state legislators to formulate relationships with their company.

This paper is an in-depth analysis of 19 e-mail chains sent between Coca-Cola representatives, lobbyists, academics, and the International Life Sciences Institute (ILSI), founded by former Coca-Cola executives. The e-mails and accompanying documents were obtained by the U.S. Right to Know (USRTK) through the Freedom of Information requests (15, 16). The e-mail chains covered the period of September 2014 to June 2015, and accompanying documents included a planning meeting itinerary from February 2014. All these documents were used in analyzing the applicability of the proposed ethics framework to emerging themes in the broader context of tangible company strategy.

The majority of correspondents on these e-mail chains were affiliated with the Global Energy Balance Network (GEBN), ILSI, and health-related departments at universities across the United States. GEBN and ILSI are prominent food and beverage industry-funded lobbying groups that generate “faux” science (17, 18). The GEBN disbanded in 2015 and no longer is in operation (19). A number of Coca-Cola employees, however, were included in these exchanges, and several messages were sent between employees and affiliates, indicating the close contact between Coca-Cola, GEBN, and ILSI. Communications were interspersed in response to: (1) news covering a 2015 International Food Information Council (IFIC) Foundation teleconference that concerned a United States Dietary Guidelines Advisory Committee advisory report, (2) the appointment of a new program head for the U.S. “Let's Move!” campaign in 2015, and (3) the prospective relationship between Coca-Cola and a state senator in West Virginia. Also included was a strategic planning agenda for a Coca-Cola meeting held in 2014 that aimed to communicate science on sugar-sweetened beverages in a manner flattering to the brand.

Using the eleven ethical principles (defined in Table 1) a codebook was assembled using these principles as codes and assigned definitions for each code from the original paper. In total, 128 instances were identified in the e-mails and a minimum of two, a maximum of eight codes were applied to respective e-mails by one author. Other authors reviewed and in the initial review 80% agreement was achieved; to resolve the dispute the reviewers discussed each code to achieve 100 percentage agreement intercoder reliability. Illustrative quotations from e-mails were designated into a matrix according to the eleven ethical codes. An inductive grounded theory was used to identify themes that originated from the quotes in these e-mails (20).

**Table 1:** Ethics framework for commercial determinants of health applied to emails (*N* = 16).

**Code**	**Definition**	**Total frequency (%)**	**No. of documents (%)**
1. Moral responsibility	Corporate actors bear moral responsibility for the harms that result from the products and services they produce and should be held responsible for actions that promote ill health	4 (3.13%)	4 (5%)
2. Non-maleficence	Corporate actors should minimize potential harm to individuals and populations from their products. Priority should be given to designing and implementing feasible interventions aimed at addressing CDoH in connection with improved health outcomes	11 (8.59%)	6 (7.5%)
3. Social justice & equity	Corporations must support fair allocation of resources as primary social goods, as well as fair distribution of the benefits and burdens associated with its products. To minimize harmful impacts, efforts should be put into protecting vulnerable groups and supporting the empowerment of disadvantaged groups	12 (9.36%)	9 (11.25%)
4. Consumer sovereignty	Individuals and communities have the right to be informed about potential harms that are associated with exposure to or consumption of a particular commodity	22 (17.19%)	13 (16.25%)
5. Evidence-informed actions	Corporate actors have an obligation to produce knowledge and engage in evidence-based decision-making to ensure investment in effective health promotion strategies	21 (16.41%)	9 (11.25%)
6. Responsiveness	The health needs of diverse populations are constantly changing. Corporations must be responsive to the dynamic needs of populations and be equipped to readily adapt their operations in line with such needs	6 (4.69%)	2 (2.5%)
7. Accountability	Corporate actors should be held accountable for the plans, behaviors, and foreseeable results of commitments that they willingly pursue in their pursuit of maximizing profits	12 (9.36%)	6 (7.5%)
8. Appropriateness	Corporate actors should produce and market commodities; and adopt actions and strategies that are reasonable and socially and culturally acceptable	8 (6.25%)	7 (8.75%)
9. Transparency	Corporate actions and rationale for decisions should be clearly communicated to the public, with opportunities for input from various interest groups	20 (15.62%)	13 (16.25%)
10. Beneficence	Corporations should implement activities and strategies aimed at benefitting society. Most of these activities are typically implemented under the corporate social responsibility programs	6 (4.69%)	6 (7.5%)
11. Holism	CDoH activities must be perceived and evaluated as part of the whole integrated network of components (actors, inputs, processes, sub-systems) comprising the public health system. Holism also requires collaboration of various actors within the system as well as with other sectors	6 (4.69%)	5 (6.25%)
Total		128	80

The paper also aggregated codes, definitions, total frequency of the code across all e-mails, and the total number of e-mails in which the code appeared (Table 1). Finally, a relevancy score was calculated for all codes by multiplying the total frequency of the codes by the total number of e-mails that the code appeared (21). The relevancy score demonstrates a normalized count for the number of times a code appeared in the documents in order to reduce bias from a code mentioned many times in one e-mail (20).

Relevance (*z*) of codes was calculated by multiplying the total frequency of codes (*n*) by the number of documents they were coded in (*m*) (21):


$$\begin{array}{l} {\text{N}}^{*}\text{M}=\text{Z} \end{array}$$

This simplified method of determining the relevancy of codes was adapted from an approach by Keller, who proposed an equation to systematically weight all information sources equally by standardizing the total number of codes across all sources (21). Keller's equation posits that a reference coded frequently across many documents has a relatively high relevancy score (*z*), whereas a reference that is coded many times within only a few documents is less relevant to the dataset as a whole; furthermore, a reference coded only a few times across many documents may also not be as relevant (21). This paper applies a quantitative approach to evaluate the relevancy of codes due to the lack of contextual clues (i.e., laughter, intonation, body language, etc.) often associated with qualitative studies. The study was reviewed by the IRB at The George Washington University and determined to fall under the category of “Not Human Subject Research” (IRB#NCR192037 dated February 4th, 2020).

## Results

The study analyzed 19 email chains and 9 documents in total—which covered 80 A-4 size pages over 2014 and 2015. The analysis of these documents revealed several ethical issues: a lack of transparency, lack of consumer sovereignty, and lack of evidence-informed actions, in particular (Table 1). The eleven principles from the framework punctuate the essence of public health and its mission to uphold disease prevention, accountability, and social justice.

### Metrics

The three principles that were violated by the emails and coded through the framework most frequently amongst the documents were consumer sovereignty (*n* = 22), evidence-informed actions (*n* = 21), and transparency (*n* = 20). Similarly, the codes violated most regularly across documents were transparency (13 out of 19 documents), consumer sovereignty (13 out of 19 documents), evidence-informed actions (9 out of 19 documents), and social justice and equity (9 out of 19 documents).

Accordingly, consumer sovereignty (*z* = 286), transparency (*z* = 260), and evidence-informed actions (*z* = 189) were the most relevant ethical principles in the data set. All eleven principles were applied at least four times throughout the documents; however, responsiveness (*z* = 12), moral responsibility (*z* = 16), and holism (*z* = 30) were the least relevant to the data set.

### Themes

In the following sections, we provide selected quotations that apply to each of the principles. We also provide an explanation for the quotations in relation to the ethical principles:

#### Moral responsibility

It is understood that corporations have a responsibility to act ethically and conduct business in a way that reduces social, economic, environmental impacts on communities where they operate (9). Coca-Cola invests massive amounts of resources toward reducing impacts from negative press or peer-reviewed research; however, they invest far less attention toward reducing inequities catalyzed by their brand (22). In an e-mail, representatives planned to persuade the WHO to change their decision on a sugar tax as opposed to working to ameliorate the negative impacts of obesity created by their company.

“*I agree that we need to do something to try and prevent WHO from taking a completely anti-food industry stance in the obesity field. This will not be easy…”*

#### Nonmaleficence

In addition to not inflicting harm, companies are ethically bound to remove harm caused by their brand on consumers, the environment, and the market (9). Large food and beverage industries employ a diverse range of strategies to promote the consumption of their products and to ensure profits for themselves and their stakeholders (23). In pursuit of their corporate interest, the interests of their customers are always placed secondary (24). One of the critical issues for public health is how these industries view children as one of their key marketing targets (25). Corporations have focused marketing strategies for children to encourage their brand identification as early as possible and to make children their loyal customers; these corporations understand that children are a substantial part of the market and have the ability to influence their parent's decisions in making brand choices (26). Similarly, Coca-Cola actively markets their products to young people, who are traditionally more susceptible to marketing ploys, which is a concern when excessive sugar intake in children has been shown to cause childhood obesity (27, 28). Coca-Cola often cites its lower-sugar alternatives as healthy options, but these drinks still far exceed the acceptable sugar level in a drink. The President of Sparkling Brands & Strategies Marketing at Coca-Cola North America addressed the audience at the Cannes Lions festival. She is cited as saying that

“*Coca-Cola aligns its marketing to the passion points of teenagers and young adults to stay relevant.”*

#### Social justice and equity

Individuals have equal rights and agency in the exchange of goods, and it is important to view individuals as rights-bearers that may bring forward critique without retribution (9). Unfortunately, Coca-Cola holds strong influence over local, state, and international decision-makers through lobbying and corporate pressure on universities. When a former Director General of the WHO made a statement supporting regulations that would restrict the consumption of sugary soft drinks, Coca-Cola launched a plan in response.

“*I am suggesting that collectively we must find a way to start a dialogue with Dr. XX. If not, she will continue to blast us with significant negative consequences on a global basis. This threat to our business is serious.”*

#### Consumer sovereignty

Consumers are rational actors who use the information they are presented to cast their economic votes accordingly (9). It is a challenge to make fiscally wise decisions when information is not presented accurately or transparently. Coca-Cola strategically directed market wide messaging and cherry-picked science from other fields, such as the exercise and fitness discipline, in lieu of evidence from peer-reviewed dietary science. This promotes a misrepresentative and incomplete depiction of the health-related risk associated with consumption of sugary beverages. When FoodMinds, a nutrition communication and consulting company, sponsored a Coca-Cola strategy meeting in 2014, Coca-Cola highlighted two of their staff as expert speakers in a meeting focused on “obesity, diabetes, and related health conditions.” One of these “expert” speakers held a degree in public administration while the other held a degree in psychology and advertising. One included in his biography:

“*[He] is very passionate about food… eating all forms and types of food… so it is rarely not on his mind.”*

#### Evidence-informed actions

Companies are obligated to contribute to the body of evidence that informs policy, investment, and health promotion activities (9). In their 2014 strategy meeting, Coca-Cola outlined an agenda that did not include evidence production, rather it was geared toward “identify[ing] other non-research approaches required,” and describing obstacles faced by sugary drinks. In the meeting introduction, their notes read:

“*Sugar-sweetened beverages (SSBs) have been characterized by some in the public health community and in academia as being a leading driver of obesity, diabetes, and related health conditions… We wish to better communicate our views regarding the data.”*

#### Responsiveness

Corporate actors must address, respond, and act promptly to public health threats in order to meet the needs of the population (9). While representatives of Coca-Cola in the e-mail threads were very responsive to threats perceived to their brand, there was no mention of the wellbeing of consumers. For example, a number of current events and news stories concerning Coca-Cola were sent via e-mail to all Coca-Cola staff or affiliates—many of these stories highlighted the health concerns attached to sugary drinks. As opposed to mitigating the problem, representatives strategized to pressurize critics:

“*Maybe we could visit her and explain about the Global Energy Balance Network and why we feel effective public-private partnerships are essential if we are going to solve the obesity problem.”*

#### Accountability

Governments must be uninfluenced by corporate actors to be able to enforce ethical practices among corporate entities (9). Therefore, when Coca-Cola and lobbying groups meet privately with decision-makers, it poses a conflict of interest. For example:

“*As the President of ILSI, I had a special and productive luncheon with the former DG, Dr. XX in 1995 at his private dining room in the WHO Geneva Headquarters to tell him about ILSI and how the two organizations could work with each other.”*

#### Appropriateness

It is vital that companies assure their products are appropriate and acceptable in the communities where they sell because consumers have rights and deserve respect and care (9). When Coca-Cola staff were organizing a campaign against a former Director General of WHO they suggested partnering with humanitarian organizations, like PEPFAR or the Bill Gates Foundation, that have positive reputations, or with US scientists who have influence, like an adjunct professor at the University of Colorado:

“*He says that WHO reports gets a lot of publicity and they must find a way of someone such as a famous scientist arrange to pay her a visit, either XX [him] or someone of similar stature or a US government scientist.”*

#### Transparency

Companies need to be transparent and promote a free flow of information for consumers and stakeholders to be able to make decisions that optimize their health (9). Coca-Cola, however, intentionally dictated a narrative around their brand, which did not maximize discourse surrounding their company's practice. For instance, in an e-mail, one Coca-Cola representative corresponded with another to share a story with a state legislator:

“*I was talking to a state senator here in WV (seems like that is all I do now) who writes a newspaper column (often about health issues)… He was very interested and I wanted to send him a copy [of the report about the F&B industry]. He informed me of the strength of TCCC lobby in WV: :-)”*

#### Beneficence

It is imperative that corporations repay consumers by working to maximize prosperity in their communities (9). It is true that Coca-Cola expressed concern for its consumers; however, most of this concern is misplaced since their actions do not align with their words. For example, obesity is mentioned 16 times throughout the e-mails, but Coca-Cola does not take ownership of the obesity problem.

“*Since WHO, as you stated has been helped by the pharmaceutical industry to combat HIV/AIDS, why not work closely with the food industry to combat obesity?”*

#### Holism

Finally, companies must account for impacts of their brand on the broader domain. One danger of Coca-Cola's marketing strategy is that it underestimated the importance of a healthy diet in maintaining physical wellbeing by transferring the responsibility to physical exercise—this is a red herring fallacy because the two are concomitantly important to achieve holistic health (29, 30). In this e-mail, representatives discuss the former Director General of WHO's stance on “Energy Balance,” which is essentially a corporate front group led by Coca-Cola funded professionals that lobbies to minimize the effect of sugary products on obesity and call for greater exercise to negate the problem. It is critical to view companies broadly and their impacts not only on the product they sell, but the other health, social, political, environmental impacts that these actions have on other areas of society, like the growing obesity epidemic.

“*Heard from others she's a food activist. We're all for healthier diets—but… Does she understand energy balance. This is where GEBN can do a lot to educate–NOT ADVOCATE–you're a 501c3. :)”*

## Discussion

Overall, this case study provided direct and detailed evidence that big food and drink companies like Coca-Cola promote deliberate and coordinated efforts to influence scientific evidence and expert opinions. These industry strategies are in violation of many ethical principles and the eleven principles identified in this study can be used to unpack the complex ethical ties between corporations and CDoH. The most relevant principles to this Coca-Cola case study were consumer sovereignty, transparency, and evidence-informed actions, which were all interconnected. Coca-Cola appeared to manipulate data, evidence, and research to craft biased messaging, which they then lobbied to consumers, policymakers, and other stakeholders, thus failing to achieve transparency, consumer sovereignty, or evidence-informed actions of industry strategies and practices and their impacts on public health.

Even principles that had the lowest relevancy scores, like responsiveness, were useful because gaps in information can be just as revealing as frequencies of it. For instance, responsiveness was coded less frequently because most of Coca-Cola's e-mails centered around responding to threats to their brand, as opposed to tackling public health hazards.

Collectively, these ethical principles outline a public health approach to CDoH particularly for LMICs. Companies with unchecked influence have the power to evade accountability and moral responsibility for their actions, which in turn allows them to exert pressure and promote harmful products in countries with less agency to counteract them (10, 17). For example, in this Coca-Cola case study, a former Director General of the WHO, was considered a sizable threat to Coca-Cola's brand. WHO accused marketers of sugary drinks of contributing to rising obesity rates amongst children in developing countries. Representatives from Coca-Cola accused WHO of being anti-U.S. corporation and speaking in defense of China. They failed to consider the appropriateness of their product in LMICs and blatantly opposed the invocation from WHO. These e-mails between Coca-Cola representatives demonstrate the usefulness of this ethics framework in reviewing the actions of corporate actors in the promotion of products that are harmful to human health. It itemizes ethical concerns for CDoH and helps to frame the entire narrative. Additionally, this case study information and the quotations provide evidence of strategies and tactics that are self-serving and rarely if ever are attentive to the company's public image and ethical responsibilities. They also prove insights into the motivations of the key leadership of Coca Cola suggesting a variety of unethical activities that are contrary to the public good. The implicit acceptance of these positions among the employees and external supporters suggests that the lack of ethical awareness is normative.

The findings in this paper are based on data from a particular time period in 2014–2015, which could differ from the state of affairs currently. While this analysis is focused on the e-mail correspondence obtained through the FOI request, we cannot confirm if these actions were executed or implemented. In this way, perspectives on the way documents should be interpreted could differ based on the recipient of information. It is possible that some of the messages in these e-mails differed from tangible action, however, several of the intentions marked in these e-mails, such as targeting adolescents through marketing ploys and building relationships with public policymakers, have been reasonably confirmed through other investigative research (23, 31).

## Conclusion

This paper showcases another case study of CDoH from an industry actor and also tests the usefulness of an ethics framework by evaluating Coca-Cola's email communications. These findings reassert the importance of monitoring CDoH for public health. These eleven ethical principles also support and help examine the growing body of literature related to CDoH and health outcomes. This set of guiding ethical principles will facilitate conversations amongst researchers, government agencies, and stakeholders when analyzing the role of ethics and CDoH of private corporations. Companies themselves may also use these guiding principles to assure ethical strategies and protocol internally.

## Data availability statement

The data analyzed in this study is subject to the following licenses/restrictions: The data for this analysis included eight documents consisting of email threads between a number of academics, International Life Sciences Institute (ILSI), Coca-Cola employees and other individuals exchanged between September 2014 and June 2015. These emails were obtained by U.S. Right to Know (USRTK) through Freedom of Information requests. Some of the documents obtained are also posted in the free, searchable industry document archives hosted by the University of California, San Francisco. We determined that there was no need to obtain informed consent since these documents are now in the public domain as a result of the Freedom of Information Request. As an additional layer of protection, we sought the approval of the GWU institutional review board before engaging in our analysis. The authors signed a data transfer and use agreement with the USRTK permitting the authors to publish or publicly present information or results relating to the Data. Additionally, as an added layer of discretion (though not required) the authors took out any names in the email exchanges reported in the article. Requests to access these datasets should be directed to https://usrtk.org/our-litigation.

## Author contributions

PN made substantial contribution to the conception and design of the study. PK contributed to analysis of data and drafting the manuscript. IB and NP have been involved in interpretation of data revising the manuscript critically for important intellectual content. AH is the senior author who critically revised the manuscript and gave the final approval of the version to be submitted for publication. All authors read and approved the final manuscript.

## Funding

Partial support from Center on Commercial Determinates of Health (CCDH), Milken Institute School of Public Health, the George Washington University.

## Conflict of interest

The authors declare that the research was conducted in the absence of any commercial or financial relationships that could be construed as a potential conflict of interest.

## Publisher's note

All claims expressed in this article are solely those of the authors and do not necessarily represent those of their affiliated organizations, or those of the publisher, the editors and the reviewers. Any product that may be evaluated in this article, or claim that may be made by its manufacturer, is not guaranteed or endorsed by the publisher.
